# Suicide risk detection using artificial intelligence: the promise of creating a benchmark dataset for research on the detection of suicide risk

**DOI:** 10.3389/fpsyt.2023.1186569

**Published:** 2023-07-24

**Authors:** Mahboobeh Parsapoor (Mah Parsa), Jacob W. Koudys, Anthony C. Ruocco

**Affiliations:** ^1^Speech and Data Science Groups, CRIM - Centre de Recherche Informatique de Montréal, Montreal, QC, Canada; ^2^Department of Psychological Clinical Science, University of Toronto, Toronto, ON, Canada; ^3^Department of Psychology, University of Toronto Scarborough Toronto, Toronto, ON, Canada

**Keywords:** artificial intelligence, deep learning algorithms, theories of suicide, machine learning algorithms, speech and text analysis for suicide, text-based suicide risk detection systems

## Abstract

Suicide is a leading cause of death that demands cross-disciplinary research efforts to develop and deploy suicide risk screening tools. Such tools, partly informed by influential suicide theories, can help identify individuals at the greatest risk of suicide and should be able to predict the transition from suicidal thoughts to suicide attempts. Advances in artificial intelligence have revolutionized the development of suicide screening tools and suicide risk detection systems. Thus, various types of AI systems, including text-based systems, have been proposed to identify individuals at risk of suicide. Although these systems have shown acceptable performance, most of them have not incorporated suicide theories in their design. Furthermore, directly applying suicide theories may be difficult because of the diversity and complexity of these theories. To address these challenges, we propose an approach to develop speech- and language-based suicide risk detection systems. We highlight the promise of establishing a benchmark textual and vocal dataset using a standardized speech and language assessment procedure, and research designs that distinguish between the risk factors for suicide attempt above and beyond those for suicidal ideation alone. The benchmark dataset could be used to develop trustworthy machine learning or deep learning-based suicide risk detection systems, ultimately constructing a foundation for vocal and textual-based suicide risk detection systems.

## 1. Introduction

Globally, suicide is a leading cause of death, especially among youth (1). Hence, it is essential to identify individuals at risk of suicide. Traditional tools for assessment of suicide risk have focused on identifying suicide risk factors such as psychiatric diagnoses, agitation, and suicidal behavior (2), but the ability of these tools to predict suicidal thoughts and behaviors using isolated suicide risk factors is only marginally better than chance (3). Thus, the use of artificial intelligence (AI) to develop accurate suicide risk assessment tools has been suggested (4–8). So far, several AI systems have been implemented to detect disorder-specific suicidal ideations in people with depression (9), or schizophrenia (10), while other AI systems have aimed to detect suicidality among social media users (11, 12). Indeed, many of these AI systems have been developed using either machine learning (ML) (13–16) or deep learning (DL) algorithms (17) trained on a variety of linguistic and acoustic features (18).

Although AI systems, particularly text-based suicide risk detection systems, have demonstrated encouraging performance, their routine integration into healthcare settings requires further evaluation and validation. The use of these text-based systems has been hampered by training data heterogeneity, inconsistent quality evaluation, the lack of comparison to standardized clinical procedures, and the absence of trustworthiness assessments. One primary step to overcome these difficulties is to create a benchmark dataset from vocal and textual samples which can be collected based on a standardized and systematic manner. Such datasets encompass observed and latent linguistic and acoustic features that might explicitly and implicitly be linked to relevant suicide-related outcomes. Using the features to train ML and DL algorithms may lead to developing vocal and textual systems that have practical utility for identifying individuals at risk of suicide. However, to our knowledge, such datasets are not currently available. Therefore, in this paper, we propose an approach for creating vocal and textual datasets from vocal samples of individuals at risk of suicide based on having a history of suicidal ideation alone versus both suicidal ideation and suicide attempt, which will permit the identification of risk factors unique to suicide attempt. We also briefly review text-based systems developed for detecting suicide risk and relevant suicide theories that have promise to inform research designs intended to identify individuals at risk for transitioning from suicide ideation to attempt. This approach can inform future research that capitalizes on current advances in AI research to improve language and speech-based suicide risk detection systems.

## 2. Text-based systems for suicide risk detection

Recent research (19, 20) has highlighted the potential of suicide risk detection systems developed using ML and DL algorithms trained on textual data extracted from social media (21), electronic health records (22), and therapy transcripts (23). Several studies have used textual data from Twitter to identify patients with suicidal ideation (24) or intent (25). These systems use natural language processing (NLP) techniques for discovering certain textual features or identifying users who follow tweets related to suicide (26). For instance, some systems categorize suicide-related phrases (e.g., cannot go on, talk to someone, overdose) into distinct classes (27) while others focus on posts about suicide-related Twitter events (28). Other studies suggest that textual parts of posts on Facebook and Instagram (14, 29–31) could be useful to develop text-based suicide risk detection systems.

Indeed Facebook developed an ML-based suicide risk detection system to detect users who might be at risk of suicide^1^, and they have built a page for users to report content related to suicide^2^. Ophir et al. (32) proposed two text-based suicide risk detection systems using DL algorithms–trained using 1,024-dimensional word embeddings obtained by Elmo–that represent effective applications of social media content. They found that the system incorporating information about personality, psychosocial factors, and psychiatric diagnosis with Facebook text predicted suicide risk better than a system reliant on text alone.

Together, these studies have demonstrated the preliminary utility of analyzing textual data from social media platforms to develop suicide screening tools, which have better predictive ability than traditional suicide screening tools (33). Furthermore, the popularity of social media among adolescents and young adults (1) ensures the availability of textual data for developing these systems. However, deploying such systems could transgress privacy, a well-elaborated issue in studies focused on using AI for social media platforms (34, 35). Moreover, integrating these systems into suicide care settings remains challenging for many reasons. First, most of the textual data sets were collected from posts of users who might not have been recruited based on having suicidal ideation or a history of attempts (33). Second, many textual datasets lack demographic, racial and geographic diversity; Third, Analyzing social media posts can cause privacy issues; Finally, any textual or vocal benchmark datasets have not been created to evaluate and validate the performance of the systems. Furthermore, it is essential to understand sources of bias inherent to social media posts–including the individuals and communities who are incidentally excluded—as this limits the trustworthiness of these systems and makes them unsuitable systems to be scaled and deployed into suicide care settings. To increase the utility of these systems and the public’s trust in their potential applications, standardized methods to acquire text and speech data may be helpful as an adjunct to existing research using social media.

## 3. A standardized method to develop vocal and textual systems for suicide risk detection

We propose a novel approach to develop speech and language systems for detecting suicide risk with the potential to enhance the burgeoning literature on suicide risk using textual materials (e.g., text from social media posts and electronic health records). Our approach uses a standardized procedure to acquire spoken language data and create a benchmark dataset to establish a pilot speech and language-based suicide detection system grounded in the ideation-to-action framework of suicide. The benchmark dataset could be used to develop ML and DL classifiers to differentiate individuals’ vocal and textual samples based on the endorsement of current and/or historical suicide phenomena (e.g., suicidal ideation, suicide attempt) or elevations in suicide-related constructs (e.g., suicide capability).

### 3.1. Creating speech and language datasets

To create vocal samples and establish a benchmark dataset from vocal and textual samples, participants may be recruited from diverse ethnic, racial, and socioeconomic statuses in accordance with national census data.

Participants would be instructed to generate speech in response to various types of language tasks, including the Picture Description Task (PDT; the PDT evaluates semantic knowledge (36) and assesses structural language skills (37)), Story Recall Task (38) (SRT; the SRT evaluates verbal short-term memory and is used to detect language difficulties), and/or Verbal Fluency Task (VFT (39); the VFT assesses language and executive functioning abilities). As a starting point, we will suggest using the “Cookie Theft Picture” (CTP),^3^ which is one of the popular pictures in the PDT of and other standardized pictures. Thus, monologue speeches from participants can be collected.

We suggest the CTP as a starting point for generating this dataset for several reasons. First, the CTP is one of the components of the Boston Diagnostic Aphasia Examination (40), which is widely used to assess speech and language functioning. There is also precedence for using the CTP for similar purposes: speech-language pathologists and other health professionals (e.g., neurologists, neuropsychologists) have employed the CTP to assess speech and language deficits associated with dementia and Alzheimer’s disease (41). Second, the DementiaBank dataset, prepared by researchers at the University of Pittsburgh’s Alzheimer Research Program, is a set of textual and vocal data samples obtained from older adults while they completed the picture description task, including the CTP description task. It has been a benchmark dataset for developing AI-powered speech and language assessments for dementia (42–44) and cognitive impairment detection (45). Thus, the Dementiabank dataset could be used as a pre-trained dataset.

Crucially, to complement the set of our vocal and textual data obtained from the CTP, researchers may be encouraged to collect more vocal data samples from a novel set of standardized pictures with greater relevance to suicide. This could include pictures from image databases such as the International Affective Picture System (46), as well as images that are nonspecific, but commonly used in clinical practice, such as the Cat Rescue (47), the Picnic Scene (48), and the Divided Attention pictures (49). Drawing from the ideation-to-action framework suicide (described below), it may be fruitful to include pictorial representations of interpersonal illustrations depicting perceived burdensomeness and thwarted belongingness that could elicit sentences or phrases pertinent to theories of the suicidal ideation. Relatedly, depictions of visual stimuli that invoke fear of death or suicide capability could help characterize content related to the transition from suicidal thoughts to suicide attempts. Potential images could include the following:

- A closeup image of someone hunched over, face lit by a computer monitor, tears falling onto their hands that rest on a keyboard.
- A wide-perspective shot of someone sitting under the shade of a tree on a sunny day, with a sweater hood pulled over their head, viewing a nearby group that is picnicking and laughing.
- A panorama of someone navigating an immense, dark hedge maze.
- A crime scene of a veiled corpse featuring a tall adjacent building.

Broadly, the set of pictures might reflect gradations in suicide-related phenomena (e.g., negative affect, thoughts of death, hopelessness, suicide capability, escape, interpersonal dilemmas). In tandem with nuanced information about current suicidality and historical suicide attempt (e.g., suicide attempt recency, ideation subtype), the structural and content-related differences elicited by pictures such as these might provide predictive value beyond that of mere categories (e.g., no history of suicidality, current suicidal ideation, current suicidal ideation with past suicide attempt).

### 3.2. Ideation-to-action framework of suicide

The ideation-to-action framework of suicide is an architecture for risk factors associated with suicide (50). Indeed, it undergirds some of the most widely cited and influential theories for suicide, including the IPT (51, 52);, Integrated Motivational-Volitional Model (53), 3-Step Theory (54), and Fluid Vulnerability Theory (55). These theories emphasize the importance of separately considering the so-called ideation and action of suicide by distinguishing factors contributing to the genesis of suicidal ideation from the transition to suicide attempt. Although the risk factors vary between theories (52, 54, 56), the genesis of suicidal ideation is often attributable to negative thoughts about self and others and/or hopelessness about the mutability of these cognitions; the transition from thoughts to action tends to involve an acquisition of capability for suicide in which the probability of one acting on their suicidal thoughts increases with ability.

Recently, the IPT has been reconceptualized in the Automatic and Controlled Antecedents of Suicidal Ideation (ACASIA) (55, 57); model. The authors of the ACASIA model employed a dual-process account to accommodate the often-eschewed automatic cognitions and associations in suicidality that are overshadowed by deliberative cognitive processes. Their model echoes some sentiments of Dombrovski and Hallquist (58) who asserted that automatic Pavlovian learning processes explain self-destructive responses to stress better than deliberative decision-making. In ACASIA, automatic processes co-occur with suicide motives and opportunity factors in the categories of close others, self, future, and capability (57). Given the reliance on reflective self-report information in most text-based suicide detection systems, grounding our approach in theories of suicide might complement existing methods for detecting risk factors, even when the text is not generated concerning prompts evocative of suicide.

### 3.3. ML or DL algorithms and features

Extracting features could be one of the primary steps in developing ML or DL -based suicide detection system. There are several types of features pertinent to language and vocal data. Python libraries such librosa and NLTK can be used to extract various linguistic features, including lexical (e.g., total number of words, Brunet’s Index, and Honor’s Statistic (59)), syntactic, semantic, and pragmatic features (60).

In terms of transcript content, it may be promising to extract words that map to various processes described in suicide theories. For instance, words like burden, alone, and hopeless may relate most to the risk of developing suicidal ideation while unafraid and painless phrases may be more strongly related to the risk of transitioning from suicidal ideation to suicide attempt. Sentiment analysis of content such as that expressing apology or feelings such as shame and guilt (61) could also be valuable. Additionally, useful acoustic features that are not explicitly related to transcript content can be extracted from participants’ voices. This could include voice activity-related features, silence-related features, and prosodic features, and it would be guided by the nascent body of research on acoustic features in suicide (60).

We propose the use of feature selection methods, such as variance threshold and minimal redundancy maximal relevance criterion, to select the most informative features. Collected features will be used to train ML or DL algorithms, serving as the basis of a pilot suicide risk detection system.

We suggest the use of support vector machines (SVMs), with linear or Gaussian kernels, as supervised ML algorithms to develop baseline models for evaluating our datasets. We suggest using SVMs for several reasons: (1) SVM-based classifiers are robust and powerful (62); (2) SVMs are popular traditional MLs for developing multimodal classifiers (62); (3) SVMs are superior to Naive Bayes and Radial Basis Function network classifiers for medical data sets (63); and (4) SVMs have been successfully used as learning algorithms of several suicide risk detection systems (e.g., (11, 17)). However, to develop accurate systems that can effectively identify individuals at risk of suicide, it will be essential to explore and compare the performance of other ML or DL algorithms trained on similar sets of linguistic and acoustic features extracted from our collected vocal and textual data.

## 4. Discussion

Voice is a rich and largely untapped source of data for identifying both linguistic and acoustic markers associated with suicidal ideation and suicide-relevant constructs. This paper describes a proposal to create a vocal and textual benchmark dataset that (a) has potential to standardize AI-based speech and language assessments; (b) encompasses observable and latent linguistic and acoustic features associated with varying suicide risk factors; and (c) can be used to train ML or DL algorithms, which could serve as the basis of a pilot automatic suicide risk detection system, offering a potentially expedient and automatic means for identifying individuals at risk of suicide. At this time, research in this area is limited by the heterogeneity of textual data samples mostly collected from social media platforms. The approach we described intends to resolve this limitation through creating textual and vocal data samples in response to the CTP and a set of standardized pictures. Then, speech and language-based suicide risk detection systems can be developed on the basis of ML and DL algorithms, trained by a set of linguistic and acoustic features extracted from the datasets.

The benchmark datasets could be used to improve the performance of current developed speech- and language-based suicide risk detection systems. We particularly encourage the development of suicide risk detection systems that combine suicide theories with supervised learning approaches may be developed using these datasets. Additionally, the feature sets may have utility for comparing clinical groups per the ideation-to-action framework. It may also validate theoretical advancements, such as the incorporation of automatic cognitive associations in ACASIA (57). Although data-driven unsupervised learning approaches may also have utility for less studied high-risk populations, we expect these methods will be initially less helpful for encouraging piloting in clinical settings. Indeed, the potentially automated nature of suicide risk detection systems offers flexible and powerful options for use in primary care settings. At this stage, such systems may flag individuals at risk, but are not yet positioned to replace clinician judgment. To use risk detection systems as adjunctive clinical tools, extensive empirical validation and refinement will be required in a variety of care settings. In particular, it would be essential to develop trustworthy and explainable suicide risk detection systems which can be easily employed by general practitioners.

Although current suicide risk detection systems can mitigate the shortcomings of clinical tools in detecting suicide risk, significant enhancements may be required to use them in care settings. Our suggested approach can be lead to develop a suicide risk detection system with the great potential to mitigate the weaknesses of clinical tools in identifying pre-crisis suicide risk as well as the limited personnel resources in mental health care. Once standardized datasets are made available, it might encourage other research groups to explore whether language and vocal content generated in typical intake/follow-ups aligns with our findings. Ultimately, we expect this will lead to an uptick in the development of trustworthy AI-based suicide risk detection systems.

In conclusion, the feature set derived from our proposed datasets, which contains both traditional and nontraditional linguistic and acoustic features of suicide risk, could contribute to the development and deployment of multi-dimensional classifiers that not only identify individuals who are at risk of suicide but could also discriminate people with suicidal ideation alone from those who have attempted suicide. By implementing this proposed approach in samples of individuals at risk for suicide along multiple risk dimensions, vocal and textual benchmark datasets could be established, which could address current challenges in developing accurate, reliable, and trustworthy suicide risk detection systems.

## Author contributions

MP and AR conceptualized the topic. MP proposed designed the structure of the manuscript, performed the literature review to write the first draft of the manuscript. MP, JK, and AR wrote the final draft of the manuscript. All authors contributed to the article and approved the submitted version.

## Funding

MP has been supported by CRIM through” Projets Patrimoine: AI for Suicide Prevention.”

## Conflict of interest

The authors declare that the research was conducted in the absence of any commercial or financial relationships that could be construed as a potential conflict of interest.

## Publisher’s note

All claims expressed in this article are solely those of the authors and do not necessarily represent those of their affiliated organizations, or those of the publisher, the editors and the reviewers. Any product that may be evaluated in this article, or claim that may be made by its manufacturer, is not guaranteed or endorsed by the publisher.

## Footnotes

^1^ https://about.fb.com/news/2018/09/inside-feed-suicide-prevention-and-ai/

^2^https://www.facebook.com/help/contact/305410456169423

^3^The CTP shows a woman with two children, a boy and a girl, in a kitchen. While the woman is drying dishes next to an overflowing sink, two children are attempting to get cookies from a jar stored in the upper cupboard of the kitchen. The boy stands on an unstable stool with his hands outstretched to the jar. The girl stands beside the stool and also has a hand outstretched, ready to receive cookies from the boy.
